# Supplementary material for: ATM regulation of IL-8 links oxidative stress to cancer cell migration and invasion
Source: eLife. 2015 Jun 1;4:e07270. doi: 10.7554/eLife.07270 (PMC4463759; doi:10.7554/eLife.07270)
Supplement: Supplementary file 2. — Statistical analysis of IL8 expression in metastasis to the indicated tissue. Univariate Cox proportional hazard regression for continuous variables (first row of table) and log-rank test of equality across strata for categorical variables (grouping of samples according to IL8 expression). DOI: http://dx.doi.org/10.7554/eLife.07270.023 [file elife07270s002.docx]

**Supplementary file 2.**

Statistical analysis of IL8 expression in clinical datasets.

**Lung**

| Patients grouped by IL8 expression | **HR** | **CI.low** | **CI.up** | **Pvalue** |
| --- | --- | --- | --- | --- |
| Expr. as cont. var. (+1SD) | 1.6400 | 1.3200 | 2.0400 | 0.0000 |
| Kaplan 2 groups High vs Low | 2.0700 | 1.3200 | 3.2700 | 0.0014 |
| Kaplan 3 groups |  |  |  | 0.1031 |
| xMed vs Low | 2.0300 | -1.0300 | 4.2400 | 0.0393 |
| xHigh vs Low | 2.2100 | -1.1700 | 5.7400 | 0.1037 |
| xHigh vs Med | 1.0900 | -1.8400 | 2.2000 | 0.8089 |

**Brain**

| Patients grouped by IL8 expression | **HR** | **CI.low** | **CI.up** | **Pvalue** |
| --- | --- | --- | --- | --- |
| Expr. as cont. var. (+1SD) | 1.3300 | -1.0900 | 1.9300 | 0.1300 |
| Kaplan 2 groups High vs Low | 1.200 | -1.6300 | 2.7000 | 0.5100 |
| Kaplan 3 groups |  |  |  | 0.3800 |
| xMed vs Low | 1.8200 | -1.8600 | 6.1400 | 0.3000 |
| xHigh vs Low | 2.5700 | -1.5600 | 10.270 | 0.1700 |
| xHigh vs Med | 1.4100 | -1.7700 | 3.5400 | 0.4800 |

**Bone**

| Patients grouped by IL8 expression | **HR** | **CI.low** | **CI.up** | **Pvalue** |
| --- | --- | --- | --- | --- |
| Expr. as cont. var. (+1SD) | 1.060 | -1.0900 | 1.2300 | 0.4300 |
| Kaplan 2 groups High vs Low | -1.010 | -1.3500 | 1.3300 | 0.9600 |
| Kaplan 3 groups |  |  |  | 0.3500 |
| xMed vs Low | 1.1000 | -1.3500 | 1.6300 | 0.6400 |
| xHigh vs Low | 3.8400 | -1.1600 | 2.2900 | 0.1700 |
| xHigh vs Med | 1.6600 | -1.1400 | 1.8800 | 0.2100 |
